# Current State‐of‐the‐Art and Future Advancements of Model‐Based Meta‐Analysis

**DOI:** 10.1002/psp4.70297

**Published:** 2026-07-12

**Authors:** Phyllis Chan, Monica Simeoni, Matthew L. Zierhut, John Maringwa, Jonathan French

**Affiliations:** ^1^ Genentech, Inc. South San Francisco California USA; ^2^ GSK Brentford UK; ^3^ Certara USA, Inc. Radnor Pennsylvania USA; ^4^ Janssen‐Cilag BV Breda the Netherlands; ^5^ Johnson & Johnson Spring House Pennsylvania USA

Meta‐analysis is a quantitative approach to combining data from multiple studies that has long been recognized as the highest level of evidence in evidence‐based medicine—it is the lens through which stakeholders can appropriately view multiple sources of evidence [1]. In recent years, model‐based meta‐analysis (MBMA) has become an integral component of the model‐informed drug development (MIDD) framework [2], due to its integrated nature, including the ability to incorporate longitudinal data and pharmacologic concepts like dose–response [3, 4].

As with traditional pairwise meta‐analysis, the main purpose of MBMA in drug development involves the estimation of a treatment effect across multiple sources of evidence; however, MBMA also aims to create a predictive model that can be used to simulate unobserved scenarios. This predictive model includes parameters that help to explain between‐study and within‐study variability; thus, data from heterogeneous sources can be readily incorporated in the same analysis. As such, MBMA leverages publicly available summary data for indirect treatment comparisons and routinely incorporates covariate effects and random effects, leading to more accurate outcome predictions [5, 6]. Because of these advantages, the most common applications of MBMA have encompassed the quantification of the competitive landscape (via a comprehensive assessment of safety and/or efficacy targets necessary for differentiation, or a target product profile (TPP)) and the prediction of the probability of successful new drug development or differentiation from other available treatments. Additionally, resulting predictive models from MBMA can be utilized to guide a variety of critical decisions, such as informing trial design (e.g., dose or patient selection), trial operating characteristics optimization, and extending market access. Indeed, in survey results published in 2024 by the Oncology Dose Optimization Working Group of the International Consortium for Innovation and Quality in Pharmaceutical Development (IQ Consortium), 56% of the 18 member companies reported using MBMA routinely in their optimal dose/schedule selection process [7].

Despite MBMA providing indispensable value in the holistic understanding of disease areas and treatment risk–benefit profiles [8], publications of MBMA methodologies and use cases remain limited, potentially hindering the expansion of its utilization and ubiquitous adoption by pharmaceutical companies [9]. Therefore, the two main objectives of this themed issue were to (1) underscore the essential role of MBMA in MIDD and (2) highlight publications that incorporated MBMA approaches, including showcasing some novel applications.

Even though the precise definition of meta‐analysis does not preclude the use of individual‐level data, most of the MBMA literature refers to analyses of aggregate‐level data. Nevertheless, because individual‐level data could provide more granular information, particularly for models with a focus on prognostic covariates (e.g., disease progression models), combining individual‐ and aggregate‐level data that are available from different studies, from proprietary and external data, could better leverage the totality of evidence. When combining individual‐ and aggregate‐level data, statistical complexities arise due to different assumptions about these two data types [10]. In this themed issue, two publications investigated methods that combine individual‐ and aggregate‐level data using MBMA, with examples in chronic obstructive pulmonary disease (COPD) from Yang et al. [11] and in rheumatoid arthritis from Pham et al. [12]. A third publication by Li et al. [13] also investigated whether covariate models developed based on aggregated literature data could accurately reflect individual patient data.

Besides featuring new methodologies for MBMA, this themed issue also included a tutorial by Bracis et al. [14], providing examples of conducting MBMA with MonolixSuite, focusing on longitudinal continuous and categorical data. The tutorial also showed diagnostic plots and statistical tests that are commonly used in meta‐analysis. With the instructions provided, this tool could allow pharmacometricians who might not be familiar with the use of R, NONMEM, or Stan in the handling of aggregate‐level data to become interested in MBMA and further expand its utilization.

The majority of published MBMA examples have focused on quantifying the efficacy and safety of chronic diseases [15]. However, in practice, a common application of MBMA is to support study design and other internal decisions within the sponsor's organization. Serrano et al. [16] investigated dynamics of placebo response in atopic dermatitis, highlighting that MBMA insights can help design shorter and less costly trials, but more importantly, with the potential to reduce patient burden by optimally switching from placebo to active treatment. MBMA can also support trial design for a treatment in a new indication by using data from a previously investigated indication with a similar pathophysiological mechanism or therapeutic target, as illustrated in Javidi et al. [17].

Asiimwe et al. [18] provided a novel application in which the authors used MBMA to assess the risk–benefit tradeoff using a clinical utility index for trastuzumab–drug conjugates, a class of drugs that require a careful balance of off‐site toxicity and on‐target efficacy. In another innovative application of MBMA, Kashihara et al. [19] correlated the more immediate vaccine‐induced immunogenicity responses with the longer termed clinical efficacy for an acute condition called respiratory syncytial virus (RSV) infection. Establishing the correlation could potentially reduce the vaccine development timelines, which is crucial for a viral disease with a high mutation rate, such as RSV. Hanan et al. [20] used MBMA to identify key predictors of hepatitis B surface antigen (HBsAg) loss, explaining a significant portion of the observed heterogeneity across trials. These findings provided a quantitative framework for understanding treatment response heterogeneity, supporting more personalized strategies.

Treatment effects in specific disease areas were examined using MBMA in several publications of this themed issue, including Chen et al. [21] and Franzese et al. [22] for non‐small cell lung cancer. Additionally, Zou et al. [23] analyzed aggregate‐level patient‐reported quality of life data from pembrolizumab oncology trials across multiple indications, and Tran et al. [24] investigated publicly available data on histological outcomes of metabolic dysfunction‐associated steatohepatitis (MASH) after pioglitazone treatment, which was previously approved for the type 2 diabetes mellitus indication.

Examples of incorporating MBMA with quantitative systems pharmacology (QSP) are illustrated in the treatment of relapsed/refractory multiple myeloma (Shah et al. [25]) and with anti‐PD‐(L)1 treatment (Johnson et al. [26]) to support drug development in oncology. This two‐pronged approach of MBMA in combination with QSP can enable a more comprehensive investigation using multiple MIDD tools, and ultimately lead to more informed decision‐making.

A few publications in this themed issue relate to work that has been recently presented in part at the American Conference on Pharmacometrics (ACoP) or the Population Approach Group Europe (PAGE) conferences. Thus, this MBMA themed issue helped to assemble a collection of the most up‐to‐date research results in a peer reviewed manner, which can be useful for knowledge exchange purposes. Training fellow pharmacometricians/statisticians and educating other functions of drug development teams on the utility of MBMA have always been a major objective of the MBMA sub‐Special Interest Group (subSIG), sponsored by the International Society of Pharmacometrics (ISoP) and the American Statistical Association (ASA). Since its inception in 2020, the MBMA subSIG has reached a mature membership with representations from industry, regulatory, and academic institutions. Additionally, the subSIG has organized multiple MBMA‐focused recorded webinars, as well as tutorials, oral and poster presentations, and panel sessions at ACoP and PAGE conferences.

This MBMA themed issue is especially timely with the current pharmacometrics focus on artificial intelligence/machine learning (AI/ML). A major strength of AI/ML is in its ability to efficiently analyze and make sense of big data [27]. There is no doubt that AI/ML will drastically improve the efficiency of the various steps of the systematic review process, which is necessary prior to performing meta‐analyses. Similarly, other modeling and simulation approaches in the MIDD toolbox could also benefit from AI/ML involvement, and the integration of AI/ML into various components of the MIDD toolbox will lead to a more efficient use of valuable data.

The importance of MBMA has been recognized by regulatory agencies around the world, including those in the United States, Japan, and China [28, 29, 30]. With the increased regulatory scrutiny on MBMA in submissions and during interactions, Pharmaceutical Research and Manufacturers of America (PhRMA) has appropriately called for a guidance document from the regulatory agencies on MBMA [31]. We hope this themed issue will help start the conversation.

## Funding

The authors have nothing to report.

## Conflicts of Interest

The authors declare no conflicts of interest.
